# Supplementary material for: Prevalence and Associated Factors of Erosive Tooth Wear among Preschool Children—A Systematic Review and Meta-Analysis
Source: Healthcare (Basel). 2022 Mar 7;10(3):491. doi: 10.3390/healthcare10030491 (PMC8953165; doi:10.3390/healthcare10030491)
Supplement: Supplementary file 1 [file healthcare-10-00491-s001.zip › healthcare-1620400-supplementary.pdf]

Supplementary S1. PRISMA checklist

Table S1. PRISMA checklist

Section andTopic	Item#	Checklist Item	Location Where Item Is Reported
		**TITLE**	
Title	1	Identify the report as a systematic review.	pg 1
		**ABSTRACT**	
Abstract	2	See the PRISMA 2020 for Abstracts checklist.	pg 2
		**INTRODUCTION**	
Rationale	3	Describe the rationale for the review in the context of existing knowledge.	pg 3-4
Objectives	4	Provide an explicit statement of the objective(s) or question(s) the review addresses.	pg 4
		**METHODS**	
Eligibility criteria	5	Specify the inclusion and exclusion criteria for the review and how studies were grouped for the syntheses.	pg 4-5
Informationsources	6	Specify all databases, registers, websites, organisations, reference lists and other sources searched or consulted to identify studies. Specify thedate when each source was last searched or consulted.	pg 5
Search strategy	7	Present the full search strategies for all databases, registers and websites, including any filters and limits used.	supp file 1
Selection process	8	Specify the methods used to decide whether a study met the inclusion criteria of the review, including how many reviewers screened each recordand each report retrieved, whether they worked independently, and if applicable, details of automation tools used in the process.	pg 5-6
Data collectionprocess	9	Specify the methods used to collect data from reports, including how many reviewers collected data from each report, whether they worked independently, any processes for obtaining or confirming data from study investigators, and if applicable, details of automation tools used in theprocess.	pg 5-7
Data items	10a	List and define all outcomes for which data were sought. Specify whether all results that were compatible with each outcome domain in eachstudy were sought (e.g. for all measures, time points, analyses), and if not, the methods used to decide which results to collect.	pg 4
	10b	List and define all other variables for which data were sought (e.g. participant and intervention characteristics, funding sources). Describe any assumptions made about any missing or unclear information.	pg 5
Study risk of biasassessment	11	Specify the methods used to assess risk of bias in the included studies, including details of the tool(s) used, how many reviewers assessed each study and whether they worked independently, and if applicable, details of automation tools used in the process.	pg 6
Effect measures	12	Specify for each outcome the effect measure(s) (e.g. risk ratio, mean difference) used in the synthesis or presentation of results.	pg 6
Synthesismethods	13a	Describe the processes used to decide which studies were eligible for each synthesis (e.g. tabulating the study intervention characteristics andcomparing against the planned groups for each synthesis (item #5)).	pg 6
	13b	Describe any methods required to prepare the data for presentation or synthesis, such as handling of missing summary statistics, or dataconversions.	pg 6
	13c	Describe any methods used to tabulate or visually display results of individual studies and syntheses.	pg 6

	13d	Describe any methods used to synthesize results and provide a rationale for the choice(s). If meta-analysis was performed, describe themodel(s), method(s) to identify the presence and extent of statistical heterogeneity, and software package(s) used.	pg 6
	13e	Describe any methods used to explore possible causes of heterogeneity among study results (e.g. subgroup analysis, meta-regression).	pg 6
	13f	Describe any sensitivity analyses conducted to assess robustness of the synthesized results.	pg 6
Reporting biasassessment	14	Describe any methods used to assess risk of bias due to missing results in a synthesis (arising from reporting biases).	pg 6-7
Certainty assessment	15	Describe any methods used to assess certainty (or confidence) in the body of evidence for an outcome.	pg 7
**RESULTS**			
Study selection	16a	Describe the results of the search and selection process, from the number of records identified in the search to the number of studies included inthe review, ideally using a flow diagram.	pg 7
	16b	Cite studies that might appear to meet the inclusion criteria, but which were excluded, and explain why they were excluded.	supp file 2
Study characteristics	17	Cite each included study and present its characteristics.	pg 7, Table 1
Risk of bias instudies	18	Present assessments of risk of bias for each included study.	pg 8-10
Results of individual studies	19	For all outcomes, present, for each study: (a) summary statistics for each group (where appropriate) and (b) an effect estimate and its precision(e.g. confidence/credible interval), ideally using structured tables or plots.	Table 1
Results ofsyntheses	20a	For each synthesis, briefly summarise the characteristics and risk of bias among contributing studies.	pg 7
	20b	Present results of all statistical syntheses conducted. If meta-analysis was done, present for each the summary estimate and its precision (e.g.confidence/credible interval) and measures of statistical heterogeneity. If comparing groups, describe the direction of the effect.	supp file 3
	20c	Present results of all investigations of possible causes of heterogeneity among study results.	pg 10-12
	20d	Present results of all sensitivity analyses conducted to assess the robustness of the synthesized results.	pg 10
Reporting biases	21	Present assessments of risk of bias due to missing results (arising from reporting biases) for each synthesis assessed.	pg 8-10, fig 2
Certainty ofevidence	22	Present assessments of certainty (or confidence) in the body of evidence for each outcome assessed.	Table 4
**DISCUSSION**			
Discussion	23a	Provide a general interpretation of the results in the context of other evidence.	pg 16
	23b	Discuss any limitations of the evidence included in the review.	pg18-19
	23c	Discuss any limitations of the review processes used.	pg18-19
	23d	Discuss implications of the results for practice, policy, and future research.	pg 16-19
**OTHER INFORMATION**			
Registration andprotocol	24a	Provide registration information for the review, including register name and registration number, or state that the review was not registered.	pg 4
	24b	Indicate where the review protocol can be accessed, or state that a protocol was not prepared.	pg 4
	24c	Describe and explain any amendments to information provided at registration or in the protocol.	N/A
Support	25	Describe sources of financial or non-financial support for the review, and the role of the funders or sponsors in the review.	pg 19

Competinginterests	26	Declare any competing interests of review authors.	pg 19
Availability of data, code andother materi- als	27	Report which of the following are publicly available and where they can be found: template data collec- tion forms; data extracted from includedstudies; data used for all analyses; analytic code; any other mate- rials used in the review.	pg 19

From: Page MJ, McKenzie JE, Bossuyt PM, Boutron I, Hoffmann TC, Mulrow CD, et al. The PRISMA 2020 statement: an updated guideline for reporting systematic reviews. BMJ 2021;372:n71. doi: 10.1136/bmj.n71.For more information, visit: http://www.prisma-statement.org/

Supplemenatary S2. Search Strategy

Research title: Prevalence and Associated Risk Factors of Erosive Tooth Wear among Children - A Systematic Review and Meta-Analysis

Medline (via Ovid) Ovid MEDLINE(R) and Epub Ahead of Print, In-Process & Other Non-Indexed Citations, Daily and Versions(R) 1946 to July 13, 2020

Searched on: 15 July 2020

Results: 319

**Medline**
**1. Tooth Erosion/**
2. (tooth eros* or dental eros* or acid erosion or enamel erosion or erosive tooth wear or dental erosive wear).tw.
3. or/1-2
4. prevalence/
5. risk factors/
6. Risk Assessment/
7. (prevalence or risk factor* or risk factors assessment or risk factor function or indicating factors or erosive factors or causative factors).tw.
8. or/4-7
9. Child/
10. Adolescent/
11. (children* or child or childhood or teen or teenage* or pediatric* or paediatric* or adolescen* or boys or girls or youth or youths).tw.
12. or/9-11
13. and/3,8,12

Embase

Embase Classic+Embase 1947 to 2020 July 13

Searched on: 15 July 2020

Results: 255

**Embase**
**1. (tooth eros* or dental eros* or acid erosion or enamel erosion or erosive tooth wear or dental erosive wear).tw.**
2. prevalence/
3. risk factor/
4. risk assessment/
5. (prevalence or risk factor* or risk factors assessment or risk factor function or indicating factors or erosive factors or causative factors).tw.
6. or/2-5
7. child/
8. adolescent/

9. (children* or child or childhood or teen or teenage* or pediatric* or paediatric* or adolescen* or boys or girls or youth or youths).tw.
10. or/7-9
11. and/1,6,10

Web of Science
Searched on: 15 July 2020
Results: 413
((TS=((tooth eros* or dental eros* or acid erosion or enamel erosion or erosive tooth wear or dental erosive wear))) AND (TS=((prevalence or risk factor* or risk factors assessment or risk factor function or indicating factors or erosive factors or causative factors))) AND (TS=((children* or child or childhood or teen or teenage* or pediatric* or paediatric* or adolescen* or boys or girls or youth or youths))))

Scopus
Searched on: 15 July 2020
Results: 536
((TITLE-ABS-KEY(tooth eros*) OR TITLE-ABS-KEY(dental eros*) OR TITLE-ABS-KEY(acid erosion) OR TITLE-ABS-KEY(enamel erosion) OR TITLE-ABS-KEY(erosive tooth wear) OR TITLE-ABS-KEY(dental erosive wear))) AND ((TITLE-ABS-KEY(prevalence) OR TITLE-ABS-KEY(risk factor*) OR TITLE-ABS-KEY(risk factors assessment) OR TITLE-ABS-KEY(risk factor function) OR TITLE-ABS-KEY(indicating factors) OR TITLE-ABS-KEY(erosive factors) OR TITLE-ABS-KEY(causative factors))) AND ((TITLE-ABS-KEY(children*) OR TITLE-ABS-KEY(child) OR TITLE-ABS-KEY(childhood) OR TITLE-ABS-KEY(teen) OR TITLE-ABS-KEY(teenage*) OR TITLE-ABS-KEY(pediatric*) OR TITLE-ABS-KEY(paediatric*) OR TITLE-ABS-KEY(adolescen*) OR TITLE-ABS-KEY(boys) OR TITLE-ABS-KEY(girls) OR TITLE-ABS-KEY(youth) OR TITLE-ABS-KEY(youths)))

Supplemenatary S3. Reasons for exclusion after full-text screening

Table S2. Reasons for exclusion

Reasons Of Exclusion	Authors	Year	Title	Journal
School children (6-12 years old)	Affshana, M. and Gheena	2015	Acid erosion of teeth in a pediatric population	Journal of Pharmaceutical Sciences and Research
School children (6-12 years old)	Aidi, H. E., E. M. Bronkhorst, M. C. Huysmans and G. J. Truin	2011	Factors associated with the incidence of erosive wear in upper incisors and lower first molars: a multifactorial approach	Journal of Dentistry
No English full text	Akhmedov, A. B., M. K. Ishanova, M. T. Qodirova, E. Dosmukhamedov and I. Z. Utesheva	2020	Prevalence, prophylaxis and treatment principles of primary teeth erosion in children	International Journal of Psychosocial Rehabilitation
Adolescents (13-18 years old)	Al-Dlaigan, Y. H., L. Shaw and A. J. Smith	2002	Is there a relationship between asthma and dental erosion? A case control study	International Journal of Paediatric Dentistry
School children (6-12 years old)	Al-Malik, M. I., R. D. Holt and R. Bedi	2002	Erosion, caries and rampant caries in preschool children in Jeddah, Saudi Arabia	Community Dentistry & Oral Epidemiology
School children (6-12 years old)	Alvarez Loureiro, L., A. Fabruccini Fager, L. S. Alves, R. Alvarez Vaz and M. Maltz	2015	Erosive tooth wear among 12-year-old schoolchildren: a population-based cross-sectional study in Montevideo, Uruguay	Caries Research
School children (6-12 years old)	Alves, L. S., C. D. Brusius, N. Dame-Teixeira, M. Maltz and C. Susin	2015	Dental erosion among 12-year-old schoolchildren: a population-based cross-sectional study in South Brazil	International Dental Journal
School children (6-12 years old)	Arnadottir, I. B., W. P. Holbrook, H. Eggertsson, H. Gudmundsdottir, S. H. Jonsson, J. O. Gudlaugsson, S. R. Saemundsson, S. T. Eliasson and H. Agustsdottir	2010	Prevalence of dental erosion in children: a national survey	Community Dentistry & Oral Epidemiology
Adolescents (13-18 years old)	Auad, S. M., P. J. Waterhouse, J. H. Nunn and P. J. Moynihan	2009	Dental caries and its association with sociodemographics, erosion, and diet in schoolchildren from southeast Brazil	Pediatric Dentistry
Tooth wear other than erosion	Ayers, K. M., B. K. Drummond, W. M. Thomson and J. A. Kieser	2002	Risk indicators for tooth wear in New Zealand school children	International Dental Journal
Adolescents (13-18 years old)	Bartlett, D. W., P. Y. Coward, C. Nikkah and R. F. Wilson	1998	The prevalence of tooth wear in a cluster sample of adolescent schoolchildren and its relationship with potential explanatory factors	British Dental Journal

No full text in English	Bax-Adamowicz, A., A. Breborowicz and M. Borysewicz-Lewicka	2014	Oral cavity complaints reported by asthmatic children	Alergia Astma Immunologia
School children (6-12 years old)	Brusius, C. D., L. S. Alves, C. Susin and M. Maltz	2018	Dental erosion among South Brazilian adolescents: A 2.5-year longitudinal study	Community Dentistry & Oral Epidemiology
School children (6-12 years old)	Caglar, E., B. Kargul, I. Tanboga and A. Lussi	2005	Dental erosion among children in an Istanbul public school	Journal of Dentistry for Children (Chicago, Ill.)
School children (6-12 years old)	Caglar, E., N. Sandalli, N. Panagiotou, K. Tonguc and O. O. Kuscu	2011	Prevalence of dental erosion in Greek minority school children in Istanbul	European Archives of Paediatric Dentistry: Official Journal of the European Academy of Paediatric Dentistry
Review	Chadwick, B. L., D. A. White, A. J. Morris, D. Evans and N. B. Pitts	2006	Non-carious tooth conditions in children in the UK, 2003	British Dental Journal
Adolescents (13-18 years old)	Chadwick, R. G., H. L. Mitchell, S. L. Manton, S. Ward, S. Ogston and R. Brown	2005	Maxillary incisor palatal erosion: no correlation with dietary variables?	Journal of Clinical Pediatric Dentistry
No full text in English	Chen, Y. G., X. Li, D. Y. Hu, H. Shen, K. Z. Li, Y. Zhao and L. L. Peng	2009	[Prevalence of tooth erosion of 5-year-old and 12-year-old children in Xuzhou city]	Hua Xi Kou Qiang Yi Xue Za Zhi
School children (6-12 years old)	Cheng, J. and K. Campbell	2016	Caries and dental erosion: Are soroti children and adolescents at risk from increased soft-drink availability in Uganda?	African Health Sciences
School children (6-12 years old)	Chiriac, A. M., V. Mercut, D. Alexandru, S. M. Popescu, R. Mercut, F. D. Popescu, D. Luchiancenco and I. Resceanu	2019	Applications of Factorial Analysis in the Study of Risk Factors and their Chemical Influence for Erosive Dental Wear	Revista De Chimie
School children (6-12 years old)	Chiriac, A. M., V. Mercut, A. Dragos, S. M. Popescu, I. Dascalu, H. O. Manolea and I. Resceanu	2018	EPIDEMIOLOGICAL STUDY OF DENTAL WEAR IN CHILDREN BETWEEN 6 AND 11 YEARS OLD FROM DOLJ COUNTY, ROMANIA	Romanian Journal of Oral Rehabilitation
School children (6-12 years old)	Correr, G. M., R. C. Alonso, M. A. Correa, E. A. Campos, F. Baratto-Filho and R. M. Puppin-Rontani	2009	Influence of diet and salivary characteristics on the prevalence of dental erosion among 12-year-old schoolchildren	Journal of Dentistry for Children (Chicago, Ill.)
No full text in English	Cukovic-Bagic, I., J. Dumancic, M. Kujundzic-Tiljak, T. Skrinjaric and H. S. Prpic	2010	DENTAL EROSION AND ASSOCIATED RISK FACTORS AMONG PRESCHOOL CHILDREN	Paediatria Croatica
Adolescents (13-18 years old)	Dahshan, A., H. Patel, J. Delaney, A. Wuerth, R. Thomas and V. Tolia	2002	Gastroesophageal reflux disease and dental erosion in children	Journal of Pediatrics
Tooth wear other than erosion	de Andrade, F. J. P., A. D. Sales-Peres, P. G. de Moura-Grec, M.	2016	Nutritional status, tooth wear and quality of life in Brazilian schoolchildren	Public Health Nutrition

	A. A. Mapengo, A. Sales-Peres and S. H. D. Sales-Peres			
Adolescents (13-18 years old)	Deery, C., M. L. Wagner, C. Longbottom, R. Simon and Z. J. Nugent	2000	The prevalence of dental erosion in a United States and a United Kingdom sample of adolescents	Pediatric Dentistry
School children (6-12 years old)	Dugmore, C. R. and W. P. Rock	2003	The progression of tooth erosion in a cohort of adolescents of mixed ethnicity	International Journal of Paediatric Dentistry
School children (6-12 years old)	Dugmore, C. R. and W. P. Rock	2003	Asthma and tooth erosion. Is there an association?	International Journal of Paediatric Dentistry
School children (6-12 years old)	Dugmore, C. R. and W. P. Rock	2004	The prevalence of tooth erosion in 12-year-old children	British Dental Journal
School children (6-12 years old)	Dugmore, C. R. and W. P. Rock	2004	A multifactorial analysis of factors associated with dental erosion	British Dental Journal
School children (6-12 years old)	Dugmore, C. R. and W. P. Rock	2005	The effect of socio-economic status and ethnicity on the comparative oral health of Asian and White Caucasian 12-year-old children	Community Dental Health
School children (6-12 years old)	El Aidi, H., E. M. Bronkhorst, M. C. Huysmans and G. J. Truin	2010	Dynamics of tooth erosion in adolescents: a 3-year longitudinal study	Journal of Dentistry
School children (6-12 years old)	El Aidi, H., E. M. Bronkhorst, M. C. Huysmans and G. J. Truin	2011	Multifactorial analysis of factors associated with the incidence and progression of erosive tooth wear	Caries Research
Adolescents (13-18 years old)	El Karim, I. A., N. M. Sanhouri, N. T. Hashim and H. M. Ziada	2007	Dental erosion among 12-14 year old school children in Khartoum: a pilot study	Community Dental Health
Irrelevant	Eloot, A., J. Vanobbergen and L. Martens	2004	Oral health in asthmatic children: a dose-response study	Revue belge de médecine dentaire. Belgisch tijdschrift voor tandheelkunde
School children (6-12 years old)	Ersin, N. K., O. Oncag, G. Tumgor, S. Aydogdu and S. Hilmioglu	2006	Oral and dental manifestations of gastroesophageal reflux disease in children: a preliminary study	Pediatric Dentistry
School children (6-12 years old)	Frazao, J. B., L. G. Machado and M. C. Ferreira	2018	Dental erosion in schoolchildren and associated factors: A cross-sectional study	Journal of the Indian Society of Pedodontics & Preventive Dentistry
School children (6-12 years old)	Fung, A. and L. B. Messer	2013	Tooth wear and associated risk factors in a sample of Australian primary school children	Australian Dental Journal
Patients with GERD	Ganesh, M., A. Hertzberg, S. Nurko, H. Needleman and R. Rosen	2016	Acid Rather Than Nonacid Reflux Burden Is a Predictor of Tooth Erosion	Journal of Pediatric Gastroenterology and Nutrition
Adolescents (13-18 years old)	Ganss, C., J. Klimek and K. Giese	2001	Dental erosion in children and adolescents--a cross-sectional and longitudinal investigation using study models	Community Dentistry & Oral Epidemiology
School children (6-12 years old)	Garduno-Picazo, M. G., M. Ruiz-Ramos and M. Juarez-Lopez	2020	Dental Erosion Risk Factors in 6 to 12 Year Old children in Mexico City	Journal of Clinical Pediatric Dentistry
Tooth wear other than erosion	Gatou, T. and E. Mamai-Homata	2012	Tooth wear in the deciduous dentition of 5-7-year-old children: risk factors	Clinical Oral Investigations

Adolescents (13-18 years old)	Gonda-Domin, M., K. Lisiecka, R. Rojek, M. Mokrzycka, J. Szymanowicz and B. Glura	2013	Dental manifestations of gastroesophageal reflux disease in children	Przeglad Gastroenterologiczny
Adolescents (13-18 years old)	Gonzalez-Aragon Pineda, A. E., S. A. Borges-Yanez, M. E. Irigoyen-Camacho and A. Lussi	2019	Relationship between erosive tooth wear and beverage consumption among a group of schoolchildren in Mexico City	Clinical Oral Investigations
Tooth wear other than erosion	Goswami, U., S. O'Toole and E. Bernabe		Asthma, long-term asthma control medication and tooth wear in American adolescents and young adults	Journal of Asthma
No full text in English	Gu, Q., J. L. Zhu and D. Y. Tao	2019	Epidemiological profiles of dental erosion in 3- to 5-year-old children in Shanghai. [Chinese]	Journal of Shanghai Jiaotong University (Medical Science)
School children (6-12 years old)	Gurgel, C. V., D. Rios, M. A. Buzalaf, S. M. da Silva, J. J. Araujo, A. R. Pauletto and M. A. de Andrade Moreira Machado	2011	Dental erosion in a group of 12- and 16-year-old Brazilian schoolchildren	Pediatric Dentistry
Adolescents (13-18 years old)	Gurgel, C. V., D. Rios, T. M. de Oliveira, V. Tessarolli, F. P. Carvalho and M. A. Machado	2011	Risk factors for dental erosion in a group of 12- and 16-year-old Brazilian schoolchildren	International Journal of Paediatric Dentistry
Adolescents (13-18 years old)	Hamasha, A. A., F. I. Zawaideh and R. T. Al-Hadithy	2014	Risk indicators associated with dental erosion among Jordanian school children aged 12-14 years of age	International Journal of Paediatric Dentistry
Tooth wear other than erosion	Harding, M. A., H. P. Whelton, S. C. Shirodaria, D. M. O'Mullane and M. S. Cronin	2010	Is tooth wear in the primary dentition predictive of tooth wear in the permanent dentition? Report from a longitudinal study	Community Dental Health
Adolescents (13-18 years old)	Harlukowicz, K. and U. Kaczmarek	2017	Prevalence and determinants of extrinsic origin dental erosion among children and adolescents from Wroclaw	Dental and Medical Problems
School children (6-12 years old)	Holbrook, W. P., I. B. Arnadottir, S. O. Hlooversson, E. Arnarsdottir, S. H. Jonsson and S. R. Saemundsson	2014	The Basic Erosive Wear Examination (BEWE) applied retrospectively to two studies	Clinical Oral Investigations
No full text in English	Hou, X. M., Q. Zhang, X. C. Chen and J. D. Wang	2009	[Prevalence of dental erosion and associated drinks in 12-year-old population of Beijing]	Chung-Hua Kou Chiang i Hsueh Tsa Chih Chinese Journal of Stomatology
No full text in English	Huartamendia, R., A. Nappa and R. Queirolo	2012	Oral health problems related to the use of inhalational drugs in respiratory disorders	Odontoestomatologia
School children (6-12 years old)	Huew, R., P. Waterhouse, P. Moynihan, S. Kometa and A. Maguire	2012	Dental caries and its association with diet and dental erosion in Libyan schoolchildren	International Journal of Paediatric Dentistry
School children (6-12 years old)	Huew, R., P. J. Waterhouse, P. J. Moynihan, S. Kometa and A. Maguire	2011	Dental erosion and its association with diet in Libyan schoolchildren	European Archives of Paediatric Dentistry: Official Journal of the European Academy of Paediatric Dentistry
School children (6-12 years old)	Huew, R., P. J. Waterhouse, P. J. Moynihan and A. Maguire	2012	Dental erosion among 12 year-old Libyan schoolchildren	Community Dental Health

No full text in English	Jaeggi, T. and A. Lussi	2004	[Erosion in early school-age children]	Schweizer Monatsschrift fur Zahnmedizin
Adolescents (13-18 years old)	Jastaniyah, N., I. Al-Majed and A. Alqahtani	2019	The relationship between overweight/obesity and dental erosion among a group of Saudi children and adolescents	Indian Journal of Dental Research
Conference paper	Javadzadeh, F. and M. Rafeey	2012	Dental erosion and gastroesophageal reflux disease (GERD) in children	Archives of Disease in Childhood
Adolescents (13-18 years old)	Kazoullis, S., W. K. Seow, T. Holcombe, B. Newman and D. Ford	2007	Common dental conditions associated with dental erosion in schoolchildren in Australia	Pediatric Dentistry
School children (6-12 years old)	Kirthiga, M., P. Poornima, R. Praveen, B. Sakeena and P. Disha	2015	Dental Erosion and its Associated Factors In 11-16-Year Old School Children	Journal of Clinical Pediatric Dentistry
No English full text	Kosmowska, A.	2008	Clinical symptoms of pathological gastroesophageal reflux in infants and children under 6 years of age. [Polish]	Polski merkuriusz lekarski : organ Polskiego Towarzystwa Lekarskiego
Adolescents (13-18 years old)	Kumar, S., S. Acharya, P. Mishra, N. Debnath and R. Vasthare	2013	Prevalence and risk factors for dental erosion among 11- to 14-year-old school children in South India	Journal of Oral Science
School children (6-12 years old)	Kunzel, W., M. S. Cruz and T. Fischer	2000	Dental erosion in Cuban children associated with excessive consumption of oranges	European Journal of Oral Sciences
Adolescents (13-18 years old)	Larsen, M. J., S. Poulsen and I. Hansen	2005	Erosion of the teeth: prevalence and distribution in a group of Danish school children	European Journal of Paediatric Dentistry
School children (6-12 years old)	Li, J., W. Fan, Y. Lu, D. Xuan, Z. Liu, S. Huang and J. Zhang	2019	A survey on the prevalence and risk indicators of erosive tooth wear among adolescents in Guangzhou, South China	Journal of Oral Rehabilitation
Adolescents (13-18 years old)	Linnett, V., W. K. Seow, F. Connor and R. Shepherd	2002	Oral health of children with gastro-esophageal reflux disease: a controlled study	Australian Dental Journal
Adolescents (13-18 years old)	Luciano, L. C. O., M. C. Ferreira and M. A. Paschoal	2017	Prevalence and factors associated with dental erosion in individuals aged 12-30 years in a northeastern Brazilian city	Clinical Cosmetic and Investigational Dentistry
Adolescents (13-18 years old)	Mafla, A. C., X. A. Ceron-Bastidas, M. E. Munoz-Ceballos, D. C. Vallejo-Bravo and M. C. Fajardo-Santacruz	2017	Prevalence and Extrinsic Risk Factors for Dental Erosion in Adolescents	Journal of Clinical Pediatric Dentistry
School children (6-12 years old)	Maharani, D. A., S. Zhang, S. S. Gao, C. H. Chu and A. Rahardjo	2019	Dental Caries and the Erosive Tooth Wear Status of 12-Year-Old Children in Jakarta, Indonesia	International Journal of Environmental Research & Public Health [Electronic Resource]
School children (6-12 years old)	Mangueira, D. F., F. C. Sampaio and A. F. Oliveira	2009	Association between socioeconomic factors and dental erosion in Brazilian schoolchildren	Journal of Public Health Dentistry
Adolescents (13-18 years old)	Marques Martinez, L., A. M. Leyda Menendez, M. Ribelles Llop, C. Segarra Ortells, R. Aiuto and D. Garcovich	2019	Dental erosion. Etiologic factors in a sample of Valencian children and adolescents. Cross-sectional study	European Journal of Paediatric Dentistry

School children (6-12 years old)	Massignan, C., J. Moro, B. Moccelini, F. M. T. de Vasconcelos, M. Cardoso and M. Bolan	2019	Socio-economic characteristics, acid drinking patterns and gastric alterations associated with erosive tooth wear in children: a cross-sectional study	European Archives of Paediatric Dentistry: Official Journal of the European Academy of Paediatric Dentistry
Adolescents (13-18 years old)	McGuire, J., A. Szabo, S. Jackson, T. G. Bradley and C. Okunseri	2009	Erosive tooth wear among children in the United States: relationship to race/ethnicity and obesity	International Journal of Paediatric Dentistry
Duplication	Millward, A., L. Shaw and A. Smith	1994	DENTAL EROSION IN 4-YEAR-OLD CHILDREN FROM DIFFERING SOCIOECONOMIC BACKGROUNDS	Journal of Dentistry for Children
Adolescents (13-18 years old)	Monagas, J., P. Ritwik, A. Kolomensky, J. Acosta, D. Kay, L. Clendaniel and P. E. Hyman	2017	Rumination Syndrome and Dental Erosions in Children	Journal of Pediatric Gastroenterology & Nutrition
Adolescents (13-18 years old)	Mungia, R., L. A. Zarzabal, S. C. Dang, M. Baez, G. K. Stookey and J. P. Brown	2009	Epidemiologic survey of erosive tooth wear in San Antonio, Texas	Texas Dental Journal
Adolescents (13-18 years old)	Nahas Pires Correa, M. S., F. Nahas Pires Correa, J. P. Nahas Pires Correa, C. Murakami and F. M. Mendes	2011	Prevalence and associated factors of dental erosion in children and adolescents of a private dental practice	International Journal of Paediatric Dentistry
School children (6-12 years old)	Nayak, S. S., B. R. Ashokkumar, A. V. Ankola and M. Hebbal	2009	Dental erosion among 12 year old school children in belgaum city- a cross sectional study	Pakistan Paediatric Journal
School children (6-12 years old)	Nihtyanova, T., M. Kukleva, T. Miteva-Katrandzhieva, S. Petrova and A. Belcheva-Krivorova	2018	Study of the relationship between oral-hygiene habits and the presence of dental erosion in preschool and school children	Journal of IMAB - Annual Proceeding (Scientific Papers)
Adolescents (13-18 years old)	Ogunyinka, A., O. O. Dosumu and O. D. Otuyemi	2001	The pattern of toothwear amongst 12-18-year-old students in a Nigerian population	Journal of Oral Rehabilitation
Adolescents (13-18 years old)	Okunseri, C., E. Okunseri, C. Gonzalez, A. Visotcky and A. Szabo	2011	Erosive tooth wear and consumption of beverages among children in the United States	Caries Research
Adolescents (13-18 years old)	O'Sullivan, E. A. and M. E. Curzon	2000	Salivary factors affecting dental erosion in children	Caries Research
Adolescents (13-18 years old)	O'Sullivan, E. A., M. E. Curzon, G. J. Roberts, P. J. Milla and M. D. Stringer	1998	Gastroesophageal reflux in children and its relationship to erosion of primary and permanent teeth	European Journal of Oral Sciences
Adolescents (13-18 years old)	O'Sullivan, E. A. and M. E. J. Curzon	2000	A comparison of acidic dietary factors in children with and without dental erosion	Journal of Dentistry for Children
Adolescents (13-18 years old)	Padmaharish, V. and R. Abilasha	2015	Prevalence of tooth wear in children and adolescents - A survey based research	Journal of Pharmaceutical Sciences and Research
School children (6-12 years old)	Peker, S., B. Kargul, I. Tanboga, T. Tunali-Akbay, A. Yarat, F. Karakoc, R. Ersu and E. Dagli	2015	Oral health and related factors in a group of children with cystic fibrosis in Istanbul, Turkey	Nigerian Journal of Clinical Practice

School children (6-12 years old)	Peres, K. G., M. F. Armenio, M. A. Peres, J. Traebert and J. T. De Lacerda	2005	Dental erosion in 12-year-old schoolchildren: a cross-sectional study in Southern Brazil	International Journal of Paediatric Dentistry
Tooth wear other than erosion	Pineda-Higuita, S., V. Saldarriaga-Bolivar, C. Gonzalez-Penagos, S. Moreno-Callejas and A. Y. Murillo-Murillo	2019	Characteristics and severity of tooth wear in 2 to 5-year-old kindergarten children in Medellin	Acta Odontologica Latinoamericana
School children (6-12 years old)	Provatenou, E., E. G. Kaklamanos, A. Kevrekidou, I. Kosma and N. Kotsanos	2016	Erosive Tooth Wear and Related Risk Factors in 8- and 14-Year-Old Greek Children	Caries Research
School children (6-12 years old)	Rezende, G., N. M. L. Dos Santos, C. Stein, J. B. Hilgert and D. D. Faustino-Silva	2019	Asthma and oral changes in children: Associated factors in a community of southern Brazil	International Journal of Paediatric Dentistry
No full text in English	Rihter, I. D., G. Jovanov, B. Petrovic, D. Blagojevic, D. Petrovic, D. Markovic and J. Dmitrovic	2015	The Presence of Non-Carious Lesions in Children	Srpski Arhiv Za Celokupno Lekarstvo
Tooth wear other than erosion	Rios, D., A. C. Magalhaes, H. M. Honorio, M. A. Buzalaf, J. R. Lauris and M. A. Machado	2007	The prevalence of deciduous tooth wear in six-year-old children and its relationship with potential explanatory factors	Oral Health & Preventive Dentistry
Conference paper	Romano, C. and S. Cardile	2014	Gastroesophageal reflux disease and oral manifestations	Italian Journal of Pediatrics. Conference: 70th Congress of the Italian Society of Pediatrics. Palermo Italy. Conference Publication:
School children (6-12 years old)	Salas, M. M. S., F. Vargas-Ferreira, T. M. Ardenghi, K. G. Peres, M. D. Huysmans and F. F. Demarco	2017	Prevalence and Associated Factors of Tooth Erosion in 8 -12-Year-Old Brazilian Schoolchildren	Journal of Clinical Pediatric Dentistry
School children (6-12 years old)	Salas, M. M. S., F. Vargas-Ferreira, G. G. Nascimento, M. C. Huysmanns and F. F. Demarco	2018	Tooth Erosion Association with Obesity: Findings from a Brazilian Survey in Schoolchildren	Pesquisa Brasileira Em Odontopediatria E Clinica Integrada
School children (6-12 years old)	Salasa, M. M. S., R. V. F. Dantasa, H. R. Sarmento, F. Vargas-Ferreira, D. Torriani and F. F. Demarco	2014	Tooth erosion and dental caries in schoolchildren: Is there a relationship between them?	Brazilian Journal of Oral Sciences
Tooth wear other than erosion	Sales-Peres, S. H. C., A. C. Sales-Peres, J. A. Marsicano, C. A. P. Carvalho, F. S. Carvalho, J. R. P. Lauris and A. Sales-Peres	2011	The relationship between tooth wear in the primary and permanent dentitions	Community Dental Health

School children (6-12 years old)	Sales-Peres, S. H. D., S. Goya, J. J. de Araujo, A. Sales-Peres, J. R. P. Lauris and M. A. R. Buzalaf	2008	Prevalence of dental wear among 12-year-old Brazilian adolescents using a modification of the tooth wear index	Public Health
Adolescents (13-18 years old)	Sanhouri, N. M., H. M. Ziada, G. I. Ahmed and A. H. Kamis	2010	Tooth surface loss, prevalence and associated risk factors among 12-14 years school children in Khartoum State, Sudan	Community Dental Health
School children (6-12 years old)	Sarath Kumar, K. S., J. Mungara, N. R. Venumbaka, P. Vijayakumar and D. Karunakaran	2018	Oral manifestations of gastroesophageal reflux disease in children: A preliminary observational study	Journal of the Indian Society of Pedodontics & Preventive Dentistry
School children (6-12 years old)	Septalita, A., A. Bahar, A. Agustanti, A. Rahardjo, D. A. Maharani, R. Rosalien and Iop	2017	Dental erosion in 12-year-old school children living in Jakarta	1st Physics and Technologies in Medicine and Dentistry Symposium
No English full text	Seraoui, H.	2012	Nutrition and teeth erosion	Fundamental and Clinical Pharmacology
Adolescents (13-18 years old)	Shahbaz, U., F. Quadir and T. Hosein	2016	Determination of Prevalence of Dental Erosion in 12 - 14 Years School Children and Its Relationship with Dietary Habits	Jcpsp, Journal of the College of Physicians & Surgeons - Pakistan
Adolescents (13-18 years old)	Shaw, L., Y. H. al-Dlaigan and A. Smith	2000	Childhood asthma and dental erosion	Journal of Dentistry for Children
School children (6-12 years old)	Shitsuka, C., M. Correa, D. A. Duarte and M. F. Leite	2015	Quantification of Dental Biofilm in Children with Dental Erosion	Pesquisa Brasileira Em Odontopediatria E Clinica Integrada
No English full text	Silveira, E. G., M. Farias and D. R. Tames	2003	Prevalence of dental erosion in children from municipal schools in the City of Itajai (SC-Brazil)	Journal of Dental Research
Patients with special needs	Taji, S. S., W. K. Seow, G. C. Townsend and T. Holcombe	2010	A controlled study of dental erosion in 2- to 4-year-old twins	International Journal of Paediatric Dentistry
No full text in English	Truin, G. J., J. E. Frencken, J. Mulder, A. J. Kootwijk and E. Jong	2007	Prevalence of caries and dental erosion among school children in The Hague from 1996-2005. [Dutch]	Nederlands tijdschrift voor tandheelkunde
No full text in English	Truin, G. J., H. M. van Rijkom, J. Mulder and M. A. van' t Hof	2004	[Dental caries and dental erosion among 5- and 6-year old and 11- and 12-year old school children in the Hague, the Netherlands. Changing prevalences?]	Nederlands Tijdschrift voor Tandheelkunde
School children (6-12 years old)	Truin, G. J., H. M. van Rijkom, J. Mulder and M. A. van't Hof	2005	Caries trends 1996-2002 among 6- and 12-year-old children and erosive wear prevalence among 12-year-old children in The Hague	Caries Research
Adolescents (13-18 years old)	Tschammler, C., A. Simon, K. Brockmann, M. Robl and A. Wiegand	2019	Erosive tooth wear and caries experience in children and adolescents with obesity	Journal of Dentistry
School children (6-12 years old)	Vargas-Ferreira, F., J. R. Praetzel and T. M. Ardenghi	2011	Prevalence of tooth erosion and associated factors in 11-14-year-old Brazilian schoolchildren	Journal of Public Health Dentistry

Adolescents (13-18 years old)	Wang, P., H. C. Lin, J. H. Chen and H. Y. Liang	2010	The prevalence of dental erosion and associated risk factors in 12-13-year-old school children in Southern China	BMC Public Health
Adolescents (13-18 years old)	Wang, P., Y. Zhou, Y. H. Zhu and H. C. Lin	2011	Unstimulated and stimulated salivary characteristics of 12-13-year-old schoolchildren with and without dental erosion	Archives of Oral Biology
School children (6-12 years old)	Wiegand, A., J. Muller, C. Werner and T. Attin	2006	Prevalence of erosive tooth wear and associated risk factors in 2-7-year-old German kindergarten children	Oral Diseases
Adolescents (13-18 years old)	Wild, Y. K., M. B. Heyman, E. Vittinghoff, D. H. Dalal, J. M. Wojcicki, A. L. Clark, B. Rechmann and P. Rechmann	2011	Gastroesophageal reflux is not associated with dental erosion in children	Gastroenterology
Adolescents (13-18 years old)	Ximenes, R., G. Couto and E. Sougey	2010	Eating disorders in adolescents and their repercussions in oral health	International Journal of Eating Disorders
Adolescents (13-18 years old)	Yaseen, S. M., R. A. Togo, Z. Meer, A. M. Al-Dheer, M. A. Al-Futaih, A. A. Al-Jalal and N. S. Al-Qahtani	2013	Dental erosion among 12-15-year-old school boys in southern Saudi Arabia	Archives of Orofacial Science
School children (6-12 years old)	Zhang, J., Y. Du, Z. Wei, B. Tai, H. Jiang and M. Du	2015	The prevalence and risk indicators of tooth wear in 12- and 15-year-old adolescents in Central China	BMC Oral Health
School children (6-12 years old)	Zhang, S., A. M. Chau, E. C. Lo and C. H. Chu	2014	Dental caries and erosion status of 12-year-old Hong Kong children	BMC Public Health

Supplemenatary S4. Measurement index of dental erosion

Indices of dental erosion.

Index	Citation	References
Tooth wear index (TWI)	Smith and Knight (1984)	Smith, B.G.; Knight, J. K. An index for measuring the wear of teeth. *Br Dent J* **1984,** 156, 435-438.
Tooth wear index modified (TWI-modified)	Bardsley et al.	Bardsley, P.F., Taylor, S., Milosevic, A. Epidemiological studies of tooth wear and dental erosion in 14-year-old children in North West England. Part 1: The relationship with water fluoridation and social deprivation. *Br Dent J* **2004,** 197, 413-416.
Erosion Partial Recording System (EPRS)	Hasselkvist et al. (2010)	Hasselkvist, A., Johansson, A., Johansson, A.K. Dental erosion and soft drink consumption in Swedish children and adolescents and the development of a simplified erosion partial recording system. *Swed Dent J* **2010,** 34, 187-195.
O'Sullivan index	O'Sullivan (2000)	O'Sullivan, E.A. A new index for the measurement of erosion in children. *Eur J Paediatr Dent* **2000,** 1, 69-74.
Basic Erosive Wear Examination (BEWE)	Bartelett et al. (2008)	Bartlett, D., Ganss, C., Lussi, A. Basic Erosive Wear Examination (BEWE): a new scoring system for scientific and clinical needs. *Clin Oral Investig* **2008**, 12, S65-68.

Tooth wear index (TWI)

Score	Description	Teeth Evaluated	Surfaces Evaluated
0	Enamel: Surface characteristics intact<br>Dentine: intact	The whole dentition	Occlusal/incisal surfaces<br>Buccal surfaces<br>Lingual surfaces<br>Cervical surfaces<br>Whole dentition
1	Enamel: Surface characteristics lost<br>Minimal loss of contour<br>Dentine: intact		
2	Enamel: Lost<br>Dentine: <1/3 of exposed dentine<br>just exposing dentine<br>Defect <1mm		
3	Enamel: Lost<br>Dentine: <2/3 of exposed dentine<br>Substainal loss of dentine<br>Defect <1-2mm		
4	Enamel: Complete lost<br>Dentine:<br>Secondary dentin exposure with or without pulp exposure<br>Defect ≥2mm deep- pulp exposure		

TWI-modified index

Score	Description	Teeth Evaluated	Surfaces Evaluated
0	Enamel: Surface characteristics lost		Occlusal/incisal surfaces

	Minimal loss of contour<br><br>Dentine: intact		Buccal surfaces<br>Lingual surfaces<br>Cervical surfaces
1	Enamel: Lost<br><br>Dentine: <1/3 of exposed dentine<br>just exposing dentine	4 first permanent molars<br>Six upper and lower teeth	
2	Enamel: Lost<br><br>Dentine: <2/3 of exposed dentine<br>Substainal loss of dentine		
3	Enamel: Complete lost<br><br>Dentine:<br>Secondary dentin exposure with or without pulp exposure<br>Defect ≥2mm deep- pulp exposure		

Erosion Partial Recording System (EPRS)

**Score**	**Description**	**Teeth Evaluated**	**Surfaces Evaluated**
0= No erosion	No visible change<br>Developmental structures intact<br>Surface features intact	Upper and lower anterior teeth<br><br>Upper and lower molars	Occlusal/incisal surfaces<br>Buccal surfaces<br>Lingual surfaces<br>Cervical surfaces
1= Mild erosion	Smoothened enamel<br>Partial or complete loss of developing structure<br>Matt, shiny, rounded or flat enamel surface, but most surface features remained		
2= Moderate erosion	Description in Score 1<br>Changes in surface features<br>Facet or concavities within enamel<br>No dentine exposure<br>Cupping >1mm		

3=Severe erosion	Description in Score 1-2. Surface features significantly changed.<br>Dentine exposure ≤1/3<br>Cupping >1mm		
4= Very severe erosion	Dentine surface exposure >1/3 or pulp exposure<br>Fused cupping		

O'Sullivan Index

Score	Description	Teeth Evaluated	Surfaces Evaluated
0	Normal	Whole dentition	Occlusal/incisal surfaces<br>Buccal surfaces<br>Lingual surfaces<br>Cervical surfaces
1	Matt enamel surface or lack of clarity when dried		
2	Matt enamel surface or lack of clarity without drying		
3	Morphological changes localized to enamel		
4	Primary dentine exposure		
5	Reparative dentine exposure		
6	Pulpal exposure		

BEWE

Score	Description	Teeth Evaluated	Surfaces Evaluated
0	No erosive tooth wear	Highest score recorded for each of the 6 sextants	Occlusal/incisal surfaces<br>Buccal surfaces<br>Lingual surfaces<br>Cervical surfaces
1	Initial loss of enamel		
2	Distinctive defects<br>< 50% tooth structure loss<br>Involved < 50% surface area<br>Dentine is usually involved		
3	Distinctive defects<br>≥50% tooth structure loss<br>Dentine is usually involved		

Supplemenatary S5. Meta-analyses.

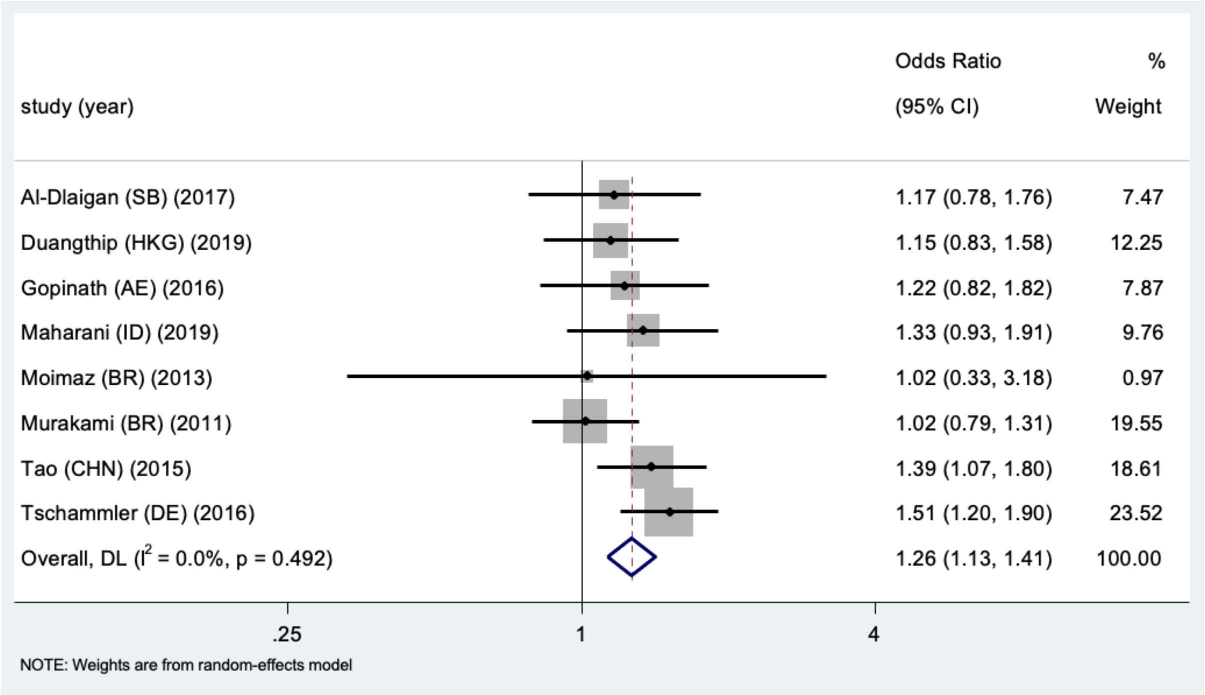

95% CI: 95% Confidence Interval; **I²**: I-square statistics**; p:** p-value

Figure S1. Forest plot showing likelihood of erosive tooth wear in boys vs girls.

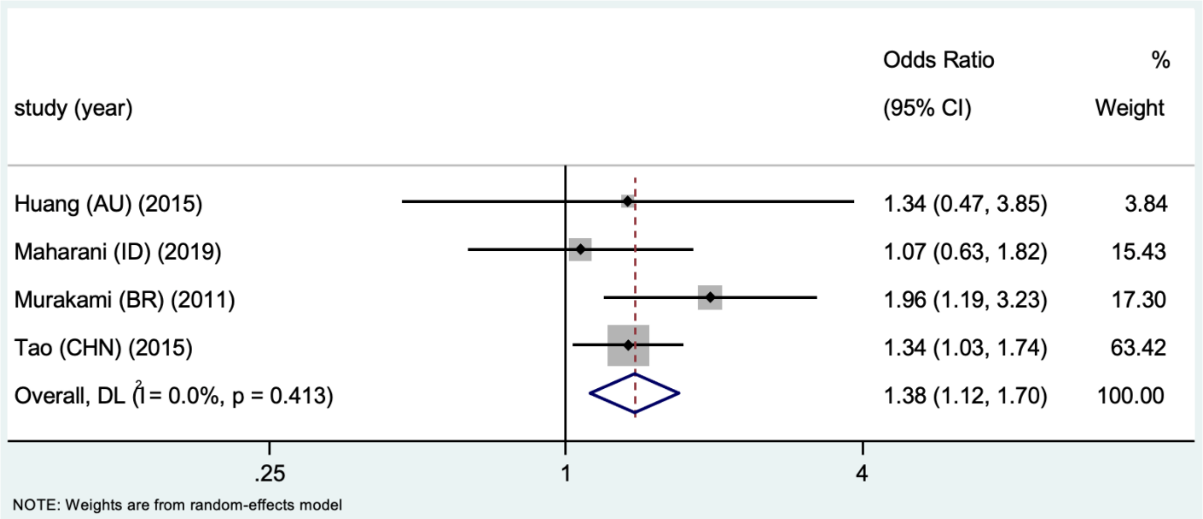

95% CI: 95% Confidence Interval; **I^2**: I-squared statistics**; p:** p-value

Figure S2. Forest plot showing likelihood of erosive tooth wear in patients with GERD, frequent vomiting, and/or digestive disorders vs patients without such digestive disorders.

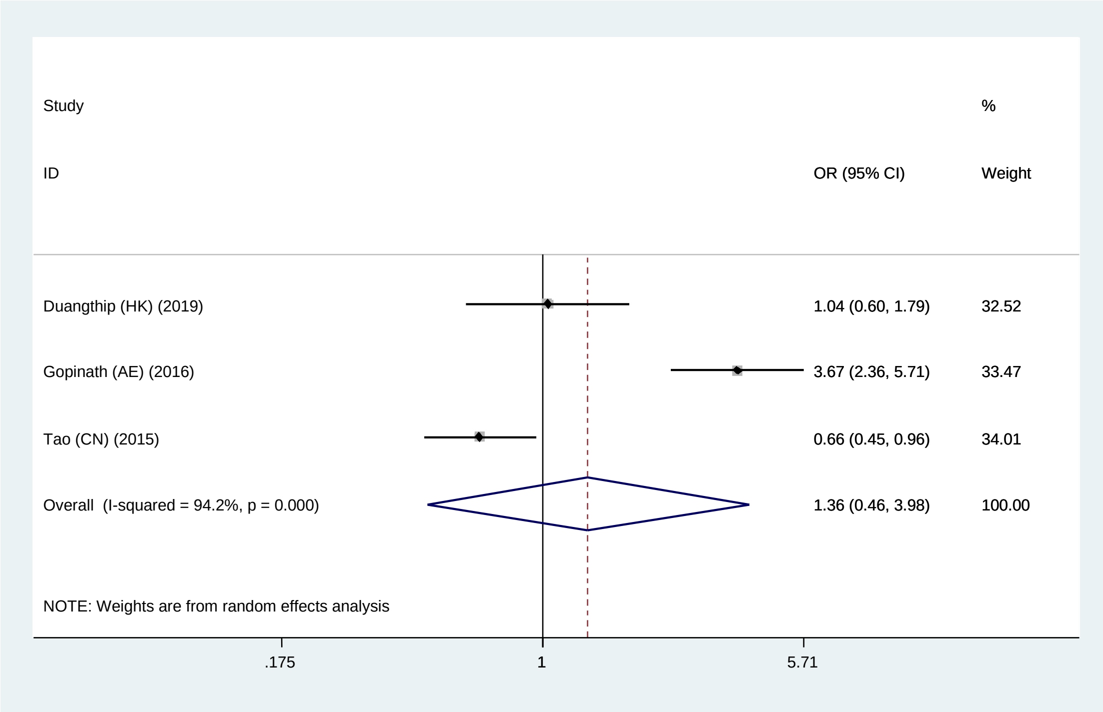

95% CI: 95% Confidence Interval; I-squared: I-squared statistics**; OR**: Odds Ratio**; p:** p-value

Figure S3. Forest plot showing likelihood of erosive tooth wear in locals vs foreigners.

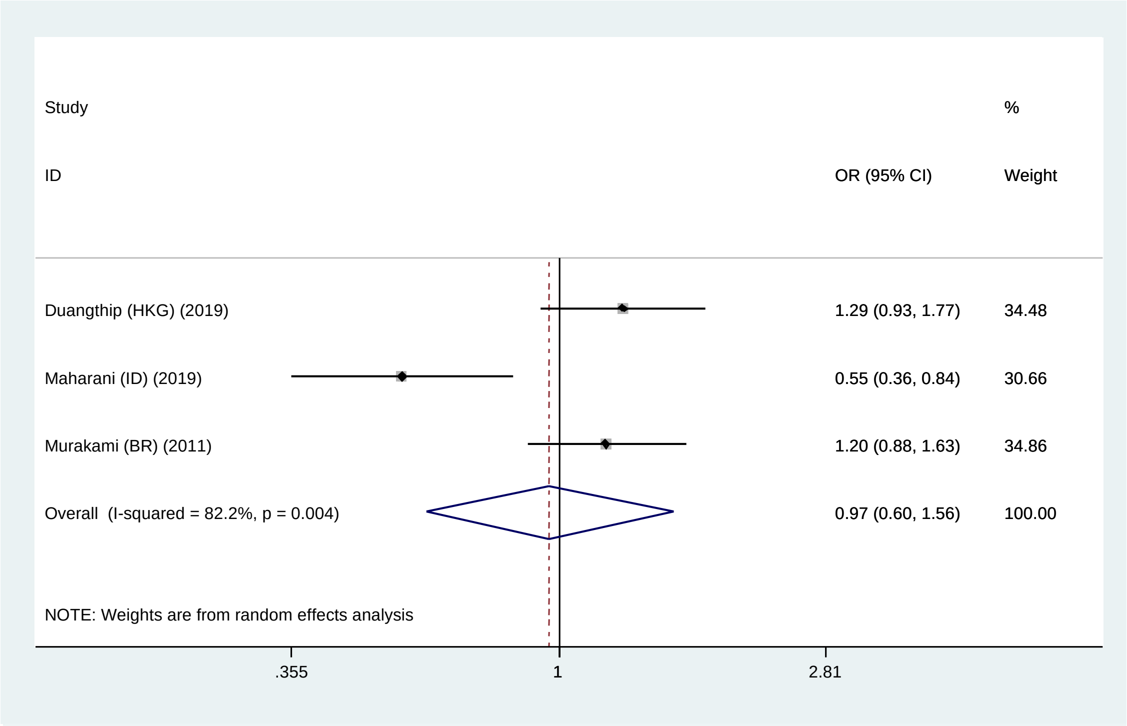

95% CI: 95% Confidence Interval; I-squared: I-squared statistics**; OR**: Odds Ratio**; p:** p-value

Figure S4. Forest plot showing likelihood of erosive tooth wear in children with caries vs without caries.

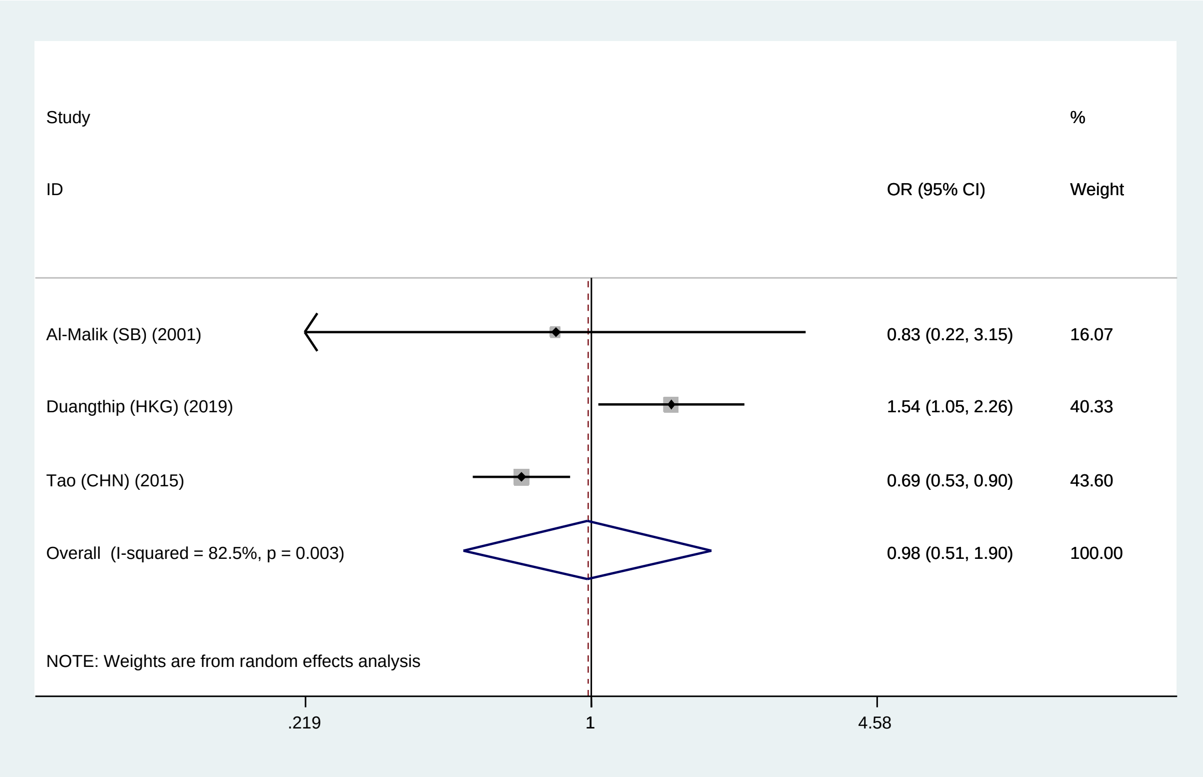

95% CI: 95% Confidence Interval; I-squared: I-squared statistics**; OR**: Odds Ratio**; p:** p-value

Figure S5. Forest plot showing likelihood of erosive tooth wear in children of parents with primary school level education and below compared to children of parents with education above primary level.

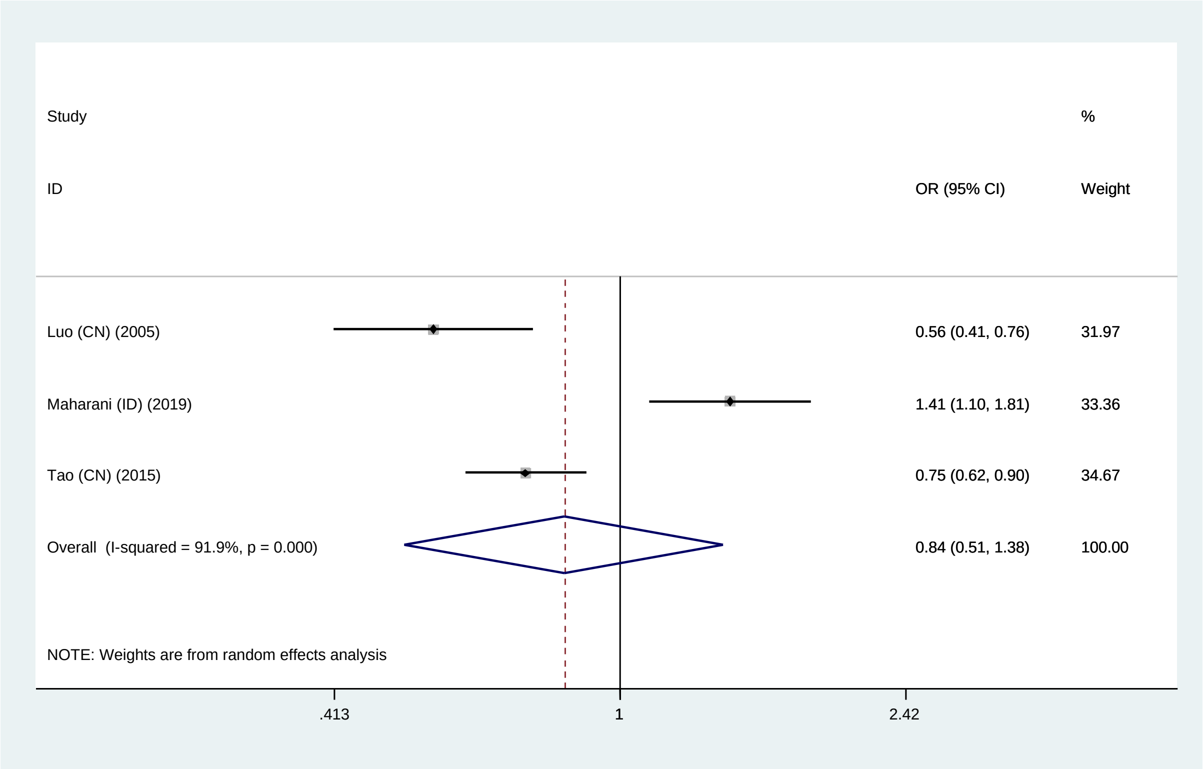

95% CI: 95% Confidence Interval; I-squared: I-squared statistics**; OR**: Odds Ratio**; p:** p-value

Figure S6. Forest plot showing likelihood of erosive tooth wear in children of parents with secondary school level education and below compared to children of parents with education above secondary level.

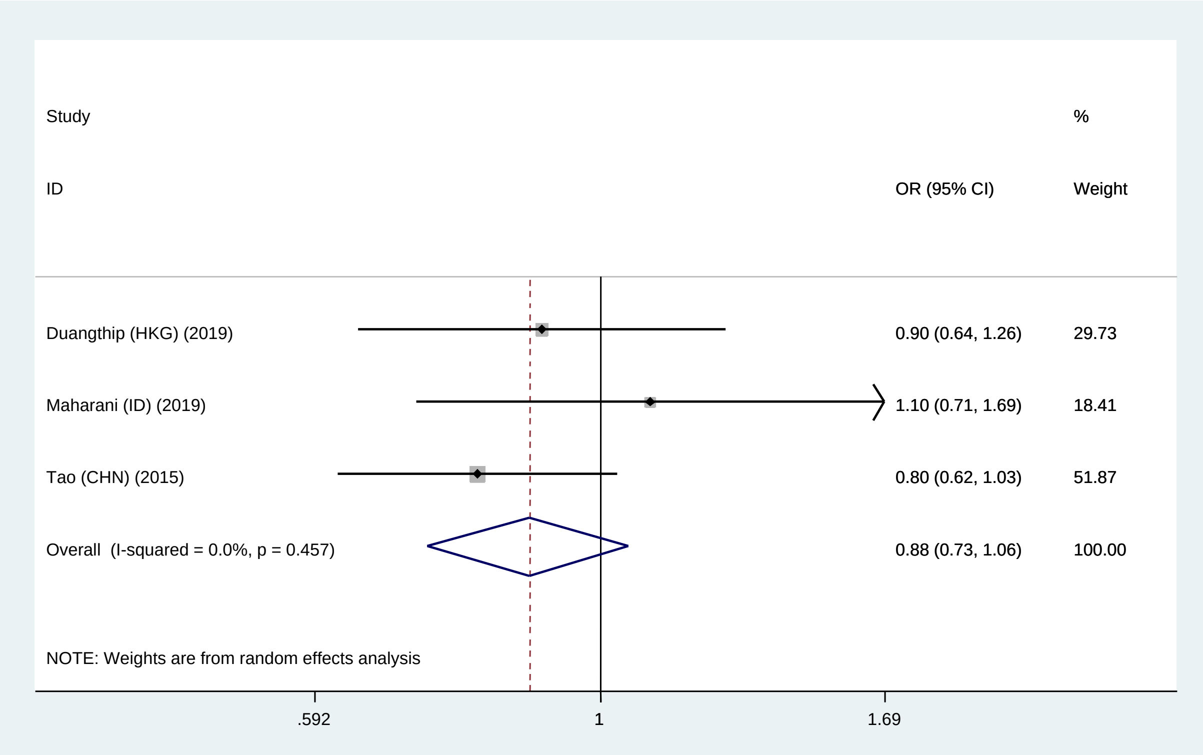

95% CI: 95% Confidence Interval; I-squared: I-squared statistics**; OR**: Odds Ratio**; p:** p-value

Figure S7. Forest plot showing likelihood of erosive tooth wear in children who brush their teeth once or less than once a day compared to children who brush their teeth more than once a day.
